# 

**Published:** 1916-09-23

**Authors:** 


### CONTENTS.

| | PAGE |
|---|---|
| **The National Mission...** | 571 |
| **An Unhappy Subscription Method** | 572 |
| **Hospital and Institutional News ...** | 573 |
| Prince Albert's Illness—Queen Mary's Hospital for the East End—The Late Sir T. Lauder Brunton—The American Red Cross and the War—The Fire at Wrest House—A Run of Legacies—The New Hospital at Muswell Hill—Extensions at Buxton—A New Convalescent Home for Merchant Seamen—A New Addition to King Edward VII.'s Hospital, Cardiff—A Hospital's Jubilee—V.A.D. Energy in Birmingham—Homes for the Disabled in Lancashire—A Curious Improvement at Cumberland Infirmary—The Nursing of Male Defectives—Malingering and Self-Mutilation—Training in Social Science and Administration—The Encouragement of Women Medical Students—The Man of Destiny—Race Suicide Encouraged at Louth—Letterkenny and Omagh Guardians Defiant—A Bogus War Charity—The Rating of an Aberdeen Hospital—A Bold Scheme of Reform—The Sympathies of Coroners' Juries—The Organisation of Hospital Sunday Parades—The Salaries of Health Visitors—An Irish Doctor's Bravery—This Week's Drug Market. | |
| **The Percy Treatment of Carcinoma of the Uterus** | 579 |
| An Ingenious Adaptation of the Cautery. | |
| **The Sphagnum-Moss Gatherers ...** | 580 |
| **Life and Death: Some Human Lessons in War-Time ...** | 581 |
| I.—The Nation and the Great War. | |
| **The School Doctor and School Feeding** | 582 |
| **Human Temperaments** | 583 |
| XI.—The Man of Action. | |
| **Two Military Hospital Gazettes ...** | 584 |
| **The Matrons' and Sisters' Department** | 585 |
| The Supply of Nurses Committee—A Slight to the Nursing Profession—The London Hospital Training School—The Supply of Nurses for the Army. | |
| Round the Hospitals. | |
| The College of Nursing—The Edinburgh Meeting—Questions for Mr. Stanley—Poor-Law Infirmary Matrons. | |
| **The Editor's Letter-Box** | 587 |
| The College of Nursing: A Glasgow View. | |
| **Legal Notes for Institutional Workers** | 588 |
| **The Heating of Hospitals ...** | 589 |
| IX.—The Birmingham General Hospital. | |
| **Matrons' and Sisters' Appointments** | 590 |
| **Gallantry at Sea** | 590 |
| **The Institutional Worker ...** (Suppl.) | 1 |
| Saving the Babies. | |

